# Real-Time Stripe Width Computation Using Back Propagation Neural Network for Adaptive Control of Line Structured Light Sensors

**DOI:** 10.3390/s20092618

**Published:** 2020-05-04

**Authors:** Jingbo Zhou, Laisheng Pan, Yuehua Li, Peng Liu, Lijian Liu

**Affiliations:** School of Mechanical Engineering, Hebei University of Science and Technology, Shijiazhuang 050018, China; zhoujingbo@hebust.edu.cn (J.Z.); panlaisheng1994@163.com (L.P.); pengliu20180702@163.com (P.L.); liulijian2002@126.com (L.L.)

**Keywords:** machine vision, line structured light sensor, back propagation neural network, stripe width computation, adaptive control

## Abstract

A line structured light sensor (LSLS) is generally constituted of a laser line projector and a camera. With the advantages of simple construction, non-contact, and high measuring speed, it is of great perspective in 3D measurement. For traditional LSLSs, the camera exposure time is usually fixed while the surface properties can be varied for different measurement tasks. This would lead to under/over exposure of the stripe images or even failure of the measurement. To avoid these undesired situations, an adaptive control method was proposed to modulate the average stripe width (ASW) within a favorite range. The ASW is first computed based on the back propagation neural network (BPNN), which can reach a high accuracy result and reduce the runtime dramatically. Then, the approximate linear relationship between the ASW and the exposure time was demonstrated via a series of experiments. Thus, a linear iteration procedure was proposed to compute the optimal camera exposure time. When the optimized exposure time is real-time adjusted, stripe images with the favorite ASW can be obtained during the whole scanning process. The smoothness of the stripe center lines and the surface integrity can be improved. A small proportion of the invalid stripe images further proves the effectiveness of the control method.

## 1. Introduction

A line structured light sensor (LSLS) is generally constituted of a laser line projector and a camera. Due to the advantages of simple construction, non-contact, and high measuring speed, it has been widely used for geometrical measurement [1,2], condition monitoring [3], profile evaluation [4], position identification [5], etc. In the measuring process, a laser plane from the projector intersects the object and a perturbed stripe image is captured by the camera. The geometrical information of the intersection profile can then be solved based on the laser triangle measurement principle [6].

Current research in LSLS mainly focuses on sensor calibration [7,8,9], stripe center extraction [10,11,12], collaborative measurement via multiple sensors [13,14,15], and the integration with motion axes [16,17,18]. The last two research scopes aim at the measurement integrity via data fusion, where the computation of the transformation matrix between different coordinate systems is the core issue. Sensor calibration is to determine the camera intrinsic parameters, lens distortion, and the equation of the laser plane [7]. For a specific LSLS, these parameters remain unchanged after the calibration. However, the stripe images, as the origin information for profile computation, are directly affected by the surface properties. However, the parts may have different geometries, colors, surface roughness, etc. Even for one specific part, its surface properties may also be varied at different regions. Undesirable stripe images will lead to poor accuracy or even failure of the measurement. A reasonable solution is to adaptively control other parameters with the surfaces accordingly.

Most adaptive control research of structured light measurement focuses on digital light processing (DLP) based fringe projection systems. Ekstrand and Zhang [19] presented a framework for automatically adjusting the image exposure by using a binary structured pattern defocusing technique where the desirable exposure time is predicted by a series of images subjected to increasing exposure times. Jiang et al. [20] fused the fringe images with different camera exposure time and projector illumination to avoid saturation and enhance the dynamic range. Feng et al. [21] divided the measured surface into several groups according to its reflectivity and obtained a better composite phase shift image by extracting the optimally exposed pixels of raw images. Song et al. [22] scanned the part several times with various camera exposure times, and then fused the multiple exposure images into one single image according to camera response function. Waddington et al. [23] presented a camera-independent method to avoid image saturation. In their method, the sinusoidal fringe patterns with different maximum gray levels are projected onto the object and the unsaturated fringe images are constructed pixel-by-pixel. Chen et al. [24] modified the projection intensity onto the part based on local surface reflectivity to avoid image saturation where the adapted fringe patterns are created prior to the measuring process. By using the DLP device, the fringe intensity can be easily adjusted at the pixel level [25,26]. This brings great flexibility and makes the above control methods possible.

The basic idea behind the above control methods is to obtain higher quality stripe images by image fusion. To achieve this goal, many raw stripe images need to be taken and the part needs to remain stationary during the measurement process. Unlike the DLP based 3D measurement technique, the LSLS has a simpler construction and a much lower cost. The laser line is generated from a semiconductor laser and shaped by a Powell lens. Its brightness can only be adjusted integrally. Moreover, the sensor always has a relative motion with the object during the scanning process. Thus, the DLP based adaptive control methods are not suited for the LSLS.

Although several methods have been developed to improve the center extraction results of poor stripe images such as the mass-spring method [27], the stripe segmentation method [28], and the multi-scale analysis method [29], they are usually complex and time consuming. Improving the quality of stripe images is still a fundamental way to guarantee a favorite measurement result. Song et al. [30] adjusted the input voltage of a laser line projector based on the wavelet decomposition and the band energy intensity. Tang et al. [31] controlled the laser intensity by using the dual-tree complex wavelet transform. These wavelet techniques involve the problem of heavy computations. Both of them are only used for selecting one optimal voltage of a batch measurement, but not for real-time control. Thus, one optimal voltage is not always suited for the whole scanning process due to the geometrical variation of the part. Wang et al. [32] adopted a reflective liquid crystal on silicon (LCoS) device to improve the image system of a laser scanner as the LCoS device could attenuate the light ray that arrived at the image pixels and avoid over exposure. Additionally, based also on the LCoS device, they further explored the adaptive control method of remedying local oversaturation [33]. The LCoS based method improved the dynamic range of the image acquisition system and the accuracy significantly. This method not only needs additional hardware, like the optical devices, LCoS device, and the control units, but also introduced a more complicate calibration process.

The motivation of our research is to enhance the sensor adaptability via real-time modulating of the average stripe width (ASW) within a desirable range. To make the method more universal, camera exposure time was selected as the control variable, which can be easily adjusted though the camera software development kit. This paper is structured as follows. First, the measurement principle is described in Section 2. In Section 3, the ASW is efficiently computed based on the back propagation neural network (BPNN). Then, the relationship between the ASW and the exposure time is discussed in Section 4. After that, a linear iterative method is proposed for exposure control in Section 5. Finally, the experiment results are analyzed and discussed in Section 6.

## 2. Measurement Principle

**Figure 1:**
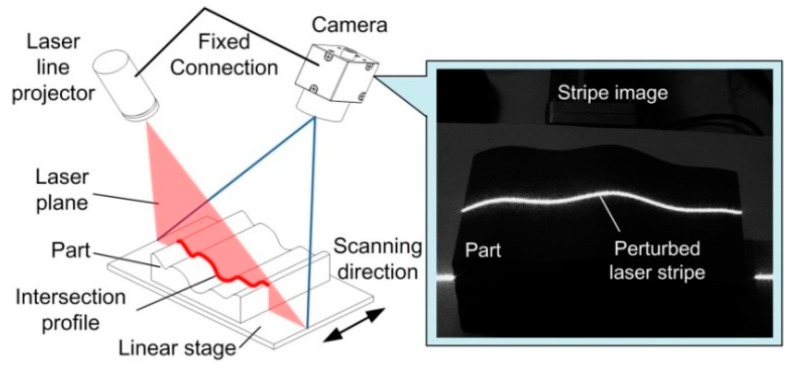
Measurement principle of the laser line scanning system.

The measurement principle of the laser line scanning system is shown in Figure 1. The laser line projector and the camera, which are fixed on the same frame, constitute the LSLS. The laser plane is emitted from the projector and intersects the part. A perturbed laser stripe reflected from the intersection profile can be captured by the camera. The stripe image carries the geometrical information. As the projector has a fixed relative position with the camera, the point coordinates on the intersection profile can be solved by the pre-calibrated sensor parameters [9]. When the part moves, the laser plane will intersect the surface at different positions and a series of intersection profiles can be achieved. The 3D topography can be obtained by combining these profiles with their translation distances [16]. In this research, we mainly focused on the adaptive control of the sensor.

## 3. Computation of ASW Using BPNN

As a measure of stripe quality, the ASW was selected as the controlled parameter. To obtain the ASW value, the first step was to compute the width of each cross section profile of the stripe image. As the stripe can be discontinuous or extremely under exposed, a minimum gray threshold value, *θ*_g_, was used to evaluate the effectiveness of each cross section profile. The width of each cross section profile was computed column by column. For one column of the stripe image, if the maximum gray value of the pixels is smaller than *θ*_g_, then it is ineffective. Only the effective cross section profiles were selected as the inputs of the BPNN.

### 3.1. Computation Principle Using BPNN

**Figure 2:**
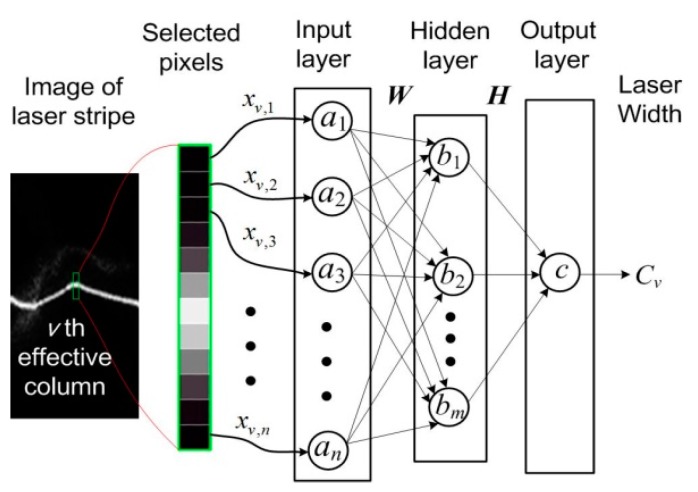
Width computation of cross section profile using back propagation neural network (BPNN).

The computation principle of each cross section profile using BPNN is shown in Figure 2. For the *v*th effective column, its central pixel is first determined by use of the extreme value method. Then, the same number of pixels is selected up and down as the network input. Neuron numbers of the input and hidden layers are *n* and *m*, respectively. The output layer only has one neuron. Network input vector is expressed by ***X****_v_* = (*x_v_*_,1_, *x_v_*_,2_, …, *x_v_*_,*n*_)^T^, and *x_v_*_,*n*_ is the normalized gray scale value of the *n*th pixel. The output vector of the input layer and hidden layer are ***A*** = (*a*_1_, *a*_2_, …, *a_n_*)^T^, ***B*** = (*b*_1_, *b*_2_, …, *b_m_*)^T^. The weight matrix from the input layer to the hidden layer is ***W****_m_*_×*n*_, where *w_i_*_,*j*_ is the value of *i*th line and *j*th column. The weight vector from the hidden layer to the output layer is the ***H***_1×*m*_, where *h_k_* is the *k*th value. *C_v_* is the output, and also the width of this column.

The weight value of input neurons is given as 1 and the activation function is *f_a_*(*x*) = *x*. Therefore, ***A*** = ***X****_v_*. The activation function of the hidden layer adopts the Sigmoid activation function, and its output vector can be expressed by

(1) $$B=\frac{1}{1+\exp(-WA)}$$

The Sigmoid activation function is also adopted for the output layer, but magnified by *n* to cover the possible width value of the cross section profile. The output for the *v*th effective column is

(2) $$C_{v}=\frac{n}{1+\exp(-HB)}$$

The ASW can be achieved by

(3) $$C_{\mathrm{avr}}=\frac{1}{V}\sum_{v=1}^{V}C_{v}$$

where *V* is the total number of effective cross sections.

### 3.2. Compute Reference Cross Section Width Using Gaussian Fitting

The reference width of each cross section profile needs to be computed for network training. As the intensity of the cross section profile follows the Gaussian distribution [34,35], Gaussian fitting (GF) can be a more robust way to access the width. The Gaussian distribution equation can be expressed by

(4) $$I_{p}=A_{0}\exp\left[-\frac{{\left(p-\mu_{0}\right)}^{2}}{2\sigma_{0}^{2}}\right]$$

where *p* is the pixel index number; *I_p_* is the gray value of this pixel; *A*_0_ is the amplitude; *μ*_0_ is the mean value; and *σ*_0_ is the standard deviation. To achieve a better fitting result, the Gauss–Newton method was adopted to solve the coefficients. Assuming *a*_0_ = *A*_0_, *a*_1_ = −1/(2$\sigma_{0}^{2}$), *a*_2_ = *μ*_0_/$\sigma_{0}^{2}$, *a*_3_ = −$\sigma_{0}^{2}$/(2$\sigma_{0}^{2}$), the fitting error for each pixel of the profile can be achieved as

(5) $$\varepsilon_{p}=I_{p}-a_{0}\exp\left(a_{1}p^{2}+a_{2}p+a_{3}\right)$$

The squared fitting error *F* can be expressed by

(6) $$F=\sum_{p=1}^{P}\varepsilon_{p}^{2}$$

where *P* is the number of the pixels for the fitting. To obtain the coefficients, the Jacobian matrix is computed as

(7) $$J=\left[\begin{array}{cccc} \frac{\partial \varepsilon_{1}}{\partial a_{0}} & \frac{\partial \varepsilon_{1}}{\partial a_{1}} & \frac{\partial \varepsilon_{1}}{\partial a_{2}} & \frac{\partial \varepsilon_{1}}{\partial a_{3}}\\ \vdots & \vdots & \vdots & \vdots \\ \frac{\partial \varepsilon_{p}}{\partial a_{0}} & \frac{\partial \varepsilon_{p}}{\partial a_{1}} & \frac{\partial \varepsilon_{p}}{\partial a_{2}} & \frac{\partial \varepsilon_{p}}{\partial a_{3}} \end{array}\right]$$

Let $\alpha ={\left(a_{0},a_{1},a_{2},a_{3}\right)}^{T}$ and $\varepsilon ={\left(\varepsilon_{1},\varepsilon_{2},\cdots ,\varepsilon_{P}\right)}^{T}$, the iterative formula can be expressed as

(8) $$\alpha^{\left(k+1\right)}=\alpha^{\left(k\right)}-{\left(J^{T}J\right)}^{-1}J^{T}\times \varepsilon^{\left(k\right)}$$

where ***α***^(*k*)^ are coefficients of the *k*th iteration and ***ε***^(*k*)^ is the corresponding fitting error. Initial coefficients, ***α***^(0)^, are calculated by the least squared method. When the deviation between current fitting error and the previous one is smaller than the given threshold value, the iteration stops.

**Figure 3:**
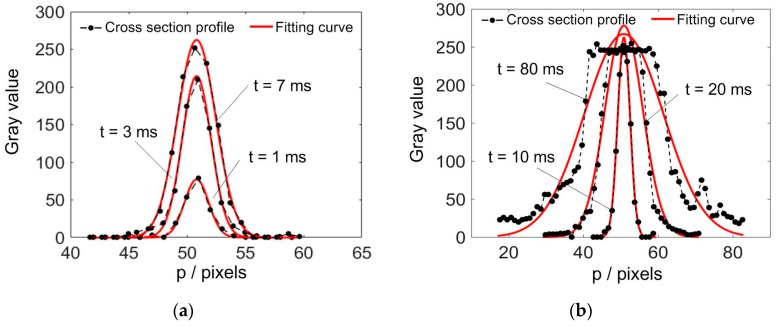
Stripe cross section profiles with different exposure time and the corresponding Gaussian fitting (GF) curves. (**a**) Unsaturated profiles, (**b**) Saturated profiles.

To verify the fitting method, cross section profiles with different exposure time were analyzed, as shown in Figure 3. Figure 3a shows three unsaturated cross section profiles. For all of the cases, the fitted curve well coincided with the profiles. When the exposure time is further increased, the stripes will become saturated. Although the fitted curves would become a little bit higher than the maximum gray value, their rising and falling edges still corresponded well with the gray values, as shown in Figure 3b. Thus, the reference width C* of the cross section profile, which is defined by 6σ_0_ of the fitting curve, can be obtained.

### 3.3. Training of BPNN

Assuming the reference width of the *v*th effective cross section profile is $C_{v}^{*}$, the squared width error between the network result and the reference value can be defined as

(9) $$E^{\left(l\right)}=\frac{1}{2}\sum_{v=1}^{V}{\left(C_{v}^{\left(l\right)}-C_{v}^{*}\right)}^{2}$$

where *l* denotes the iteration number of network training. Adjustment value of the weights can be computed according to the gradient descent principle as

(10) $$\left(\begin{array}{cc} \Delta h_{k}^{\left(l\right)}, & \Delta w_{i,j}^{\left(l\right)} \end{array}\right)=\left(\begin{array}{cc} -\eta \frac{\partial E^{\left(l\right)}}{\partial h_{k}^{\left(l\right)}}, & -\eta \frac{\partial E^{\left(l\right)}}{\partial w_{i,j}^{\left(l\right)}} \end{array}\right)\;$$

where *η* ∈ [0,1] is the factor of learning efficiency. Based on the chain rule and the activation functions, the adjustment values in Equation (10) can be expressed by

(11) $$\Delta h_{k}^{\left(l\right)}=-\eta \cdot \left(C_{v}^{\left(l\right)}-C_{v}^{*}\right)\cdot n\cdot C_{v}^{\left(l\right)}\cdot \left(1-C_{v}^{\left(l\right)}\right)\cdot b_{k}^{\left(l\right)}$$

(12) $$\Delta w_{i,j}^{\left(l\right)}=-\eta \cdot \left(C_{v}^{\left(l\right)}-C_{v}^{*}\right)\cdot n\cdot C_{v}^{\left(l\right)}\cdot \left(1-C_{v}^{\left(l\right)}\right)\cdot h_{k}^{\left(l\right)}\cdot b_{i}^{\left(l\right)}\cdot \left(1-b_{i}^{\left(l\right)}\right)\cdot a_{j}^{\left(l\right)}$$

Weights after the adjustment are

(13) $$\left(\begin{array}{cc} h_{k}^{\left(l+1\right)}, & w_{i,j}^{\left(l+1\right)} \end{array}\right)=\left(\begin{array}{cc} h_{k}^{\left(l\right)}+\Delta h_{k}^{\left(l\right)}, & w_{i,j}^{\left(l\right)}+\Delta w_{i,j}^{\left(l\right)} \end{array}\right)$$

When the training error is smaller than the given value, or the iteration has reached the maximum value, the network training is stopped. The weights of the network, **W** and **H**, can be saved.

## 4. Relationship between ASW and Exposure Time

**Figure 4:**
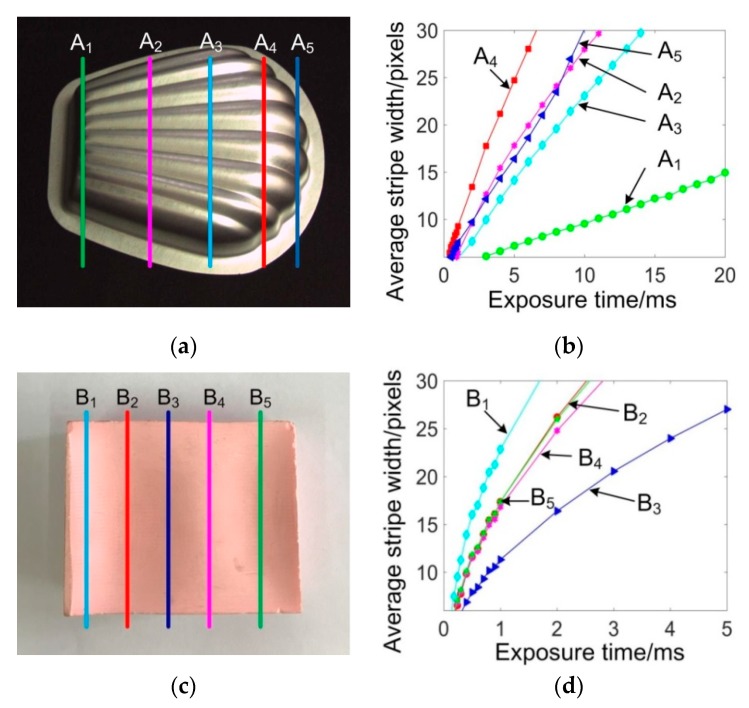
Relationship between the exposure time and ASW for parts at different intersection profiles. (**a**) Shiny part; (**b**) exposure time vs. ASW curves for intersection profiles of A1~A5; (**c**) matt part; (**d**) exposure time vs. ASW curves for intersection profiles of B1~B5.

To develop a reasonable control method, the relationship between ASW and exposure time was explored. The experiment considered different geometries, materials, and colors. Two specific parts were selected for the analysis, as shown in Figure 4. The first one, Figure 4a, had a shiny surface and the other, Figure 4c, had a matt surface. The part was placed on the stage with its surface intersecting with the laser plane at different intersection profiles. For one specific intersection profile, the exposure time was manually adjusted and the *C*_avr_ was computed by Equation (3). Figure 4b,d are the corresponding curves of the intersection profiles in Figure 4a,c, respectively. It can be seen that these curves varied remarkably, which is because the points on different profiles have different normal vectors that determine the reflective light into the camera. However, for each specific profile, the ASW increased monotonically with the exposure time.

**Figure 5:**
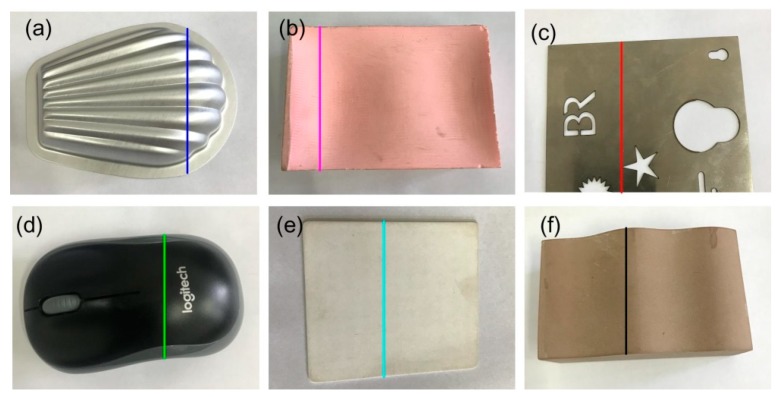
Parts of different optical reflection characters. (**a**) Aluminum stamped part; (**b**) pink freeform resin part; (**c**) stainless steel sheet; (**d**) black plastic part; (**e**) matt ceramic plate; (**f**) brown resin part.

**Figure 6:**
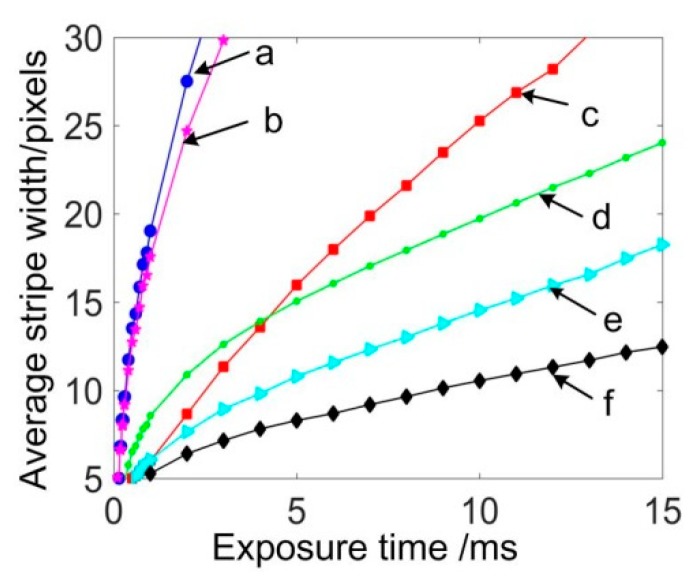
Exposure time vs. ASW curves for corresponding intersection profiles in Figure 5a–f.

To further explore the relationship, parts with different materials were examined, as shown in Figure 5. For simple analysis, one intersection profile was selected on one part. As expected, the ASW also increased with the exposure time, as shown in Figure 6. In this figure, the ASW of curve (d) increased rapidly in the early stage and then slowed down in the later stage. For other curves, there was an approximate linear relationship between the ASW and the exposure time.

From the above experiments, the following conclusions can be obtained. (1) When the camera exposure time gets longer, the ASW increases monotonically. Each exposure time vs. ASW curve is smooth and approximately follows a linear manner. (2) For a specific exposure time, the ASW can be very different under different geometries, materials, and colors. Thus, fixed sensor parameters cannot satisfy different measurement situations.

## 5. Adaptive Control of Exposure Time

**Figure 7:**
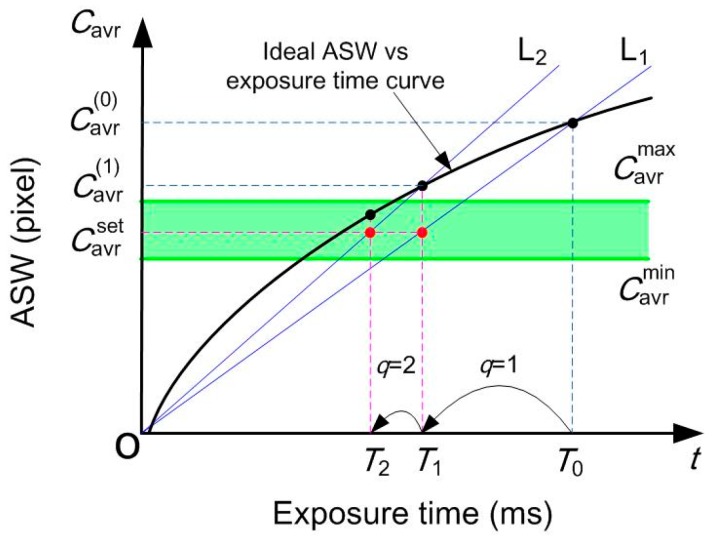
Computation principle of the linear iteration method.

Based on the above analysis, the exposure time vs. ASW curves of different intersection profiles can vary significantly, even for the same part. Thus, a linear iterative method was proposed that does not rely on the ideal exposure time vs. ASW curve, as shown by Figure 7. The control objective was to modulate the ASW within a required range of [$C_{\mathrm{avr}}^{\min}$ $C_{\mathrm{avr}}^{\max}$]. For T_0_, the stripe image is captured and the ASW is computed as $C_{\mathrm{avr}}^{\left(0\right)}$. It can be seen that $C_{\mathrm{avr}}^{\left(0\right)}$ was not within the range. Then, line L_1_ can be constructed, which also passes the origin O. The next estimated exposure time is computed by

(14) $$T_{q+1}=\frac{C_{\mathrm{avr}}^{\mathrm{set}}}{C_{\mathrm{avr}}^{\left(q\right)}}\cdot T_{q}\;q=0,1,2,\ldots ,Q$$

where *Q* is the maximum number of iterations and $C_{\mathrm{avr}}^{\mathrm{set}}$ is the ideal stripe width. After the camera exposure time is set to *T_q_*_+1_, the corresponding stripe image can be obtained and $C_{\mathrm{avr}}^{\left(q+1\right)}$ can be computed. If $C_{\mathrm{avr}}^{\left(q+1\right)}$ falls within the required range, the iteration stops. The current image is used for stripe center extraction. Otherwise, the iteration continues.

At the beginning of a measurement, the ASW under pre-defined exposure time may have a large deviation with $C_{\mathrm{avr}}^{\mathrm{set}}$. For this instance, several linear iterations may be needed. In the measuring process, the material and the color for a specific part are usually unchanged. The 3D geometry is also continuous for most of the cases. Thus, the exposure time for the next image can be estimated using the previous one.

## 6. Experiments and Analysis

**Figure 8:**
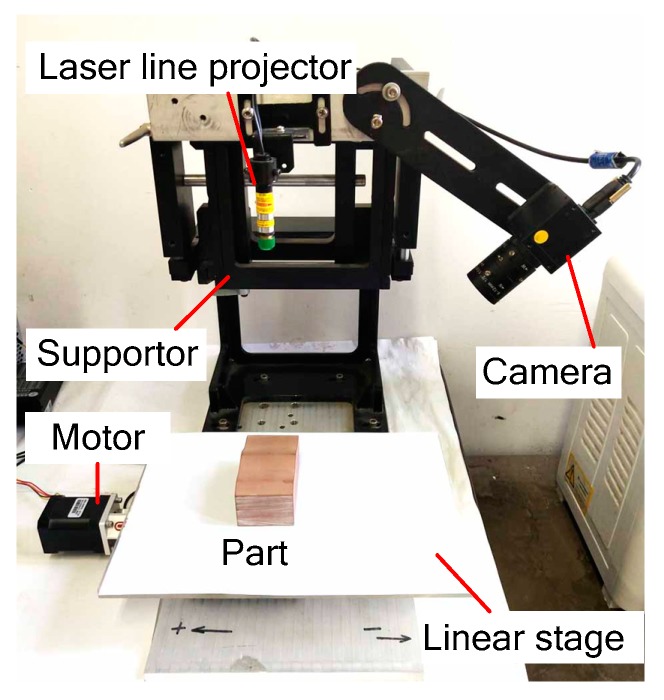
Configuration of the laser line scanning system.

The measurement system is shown in Figure 8. It generally constitutes a laser projector (Shenzuan Lasers Co. Ltd., Shantou, China), a camera (MV-UB500M, Mindvision Technology Co. Ltd., Shenzhen, China), a linear stage, and the structural parts that connect them together. The laser projector has a wavelength of 650 nm and a power of 5 mW. Its minimum line width can reach 0.4 mm at the projection distance of 300 mm. The camera resolution is 1280 × 960 pixels. The focal length of the lens is 4~12 mm, and can be manually adjusted. During the measurement process, the stripe images were processed by a computer with an Intel i5-3470 CPU and 4 GB RAM.

### 6.1. Real-Time Computation of ASW Using BPNN

**Figure 9:**
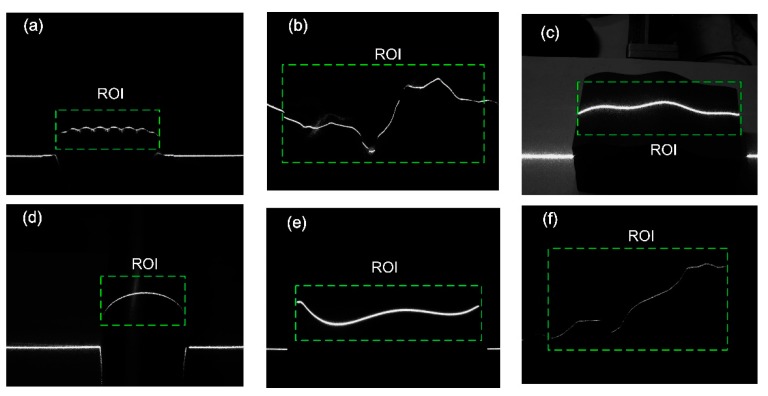
Stripes for network training and verification. (**a**) Normal exposed wave stripe; (**b**,**f**) under exposed random stripes; (**c**,**e**) over exposed smooth stripes; (**d**) normal exposed arc stripe.

The number of neurons for input and hidden layers were *n* = 31 and *m* = 13, respectively. The stripe images of Figure 9a–c were used for network training. The cross section profile widths of all selected stripes in Figure 9 were computed to verify the effectiveness of the trained network. These stripes are denoted by the region of interest (ROI) on the images. The reference width of each effective cross section profile, which is used for the network training, is computed by the use of the GF. These profiles generally cover all of the stripe status from under to over exposure. The detailed training process can be found in Section 3.3. The factor of learning efficiency is determined by experiments. When *η* = 0.5, the network converges well.

**Figure 10:**
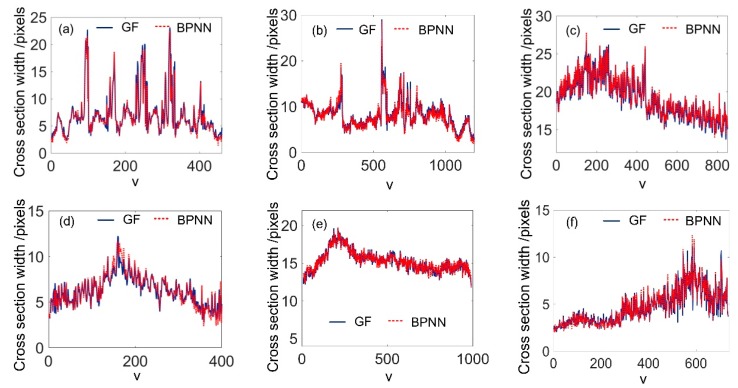
Comparison of the cross section width values by GF and BPNN, (**a**–**f**) correspond to the stripes of the ROI in Figure 9a–f.

After the training, the weights, **W** and **H**, can be obtained. The width of each cross section profile can be achieved just through the forward computation. For easy comparison, the width of each cross section profile in Figure 9 was computed by use of the GF and BPNN, respectively. The results are shown in Figure 10. It can be seen that the values obtained from the BPNN had good agreement with that of GF. The ASW values from these two methods are illustrated in Table 1. For all of the stripe images in Figure 9, the maximum deviation was 0.0403 pixels, and the minimum deviation was only 0.0048 pixels. This shows the high accuracy of the stripe width computation using BPNN. To achieve real-time exposure adjustment, the ASW should first be computed in the shortest possible time. Thus, the GF method, which needs seconds to achieve the computation, is incompetent. On the other hand, the proposed BPNN method only needs less than 1% computation time of the GF method and is more suitable.

**Figure 11:**
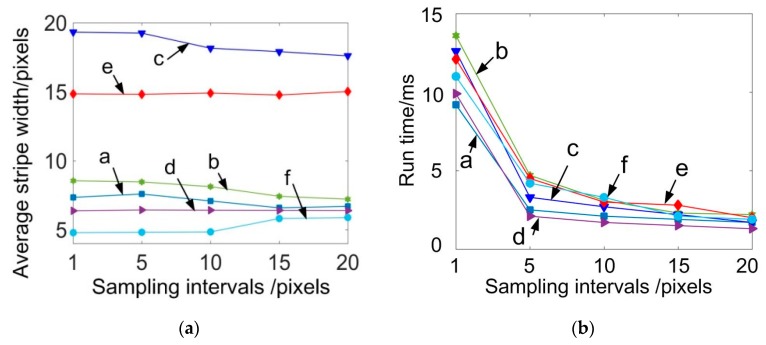
Influence of sampling intervals on the (**a**) average stripe width and the (**b**) run time.

For actual measurements, the stripes are usually continuous or piecewise continuous. To further reduce the computation time, we may not need to compute the width value of all cross section profiles, but only compute a certain amount of uniformly-sampled ones of the stripe. The sampling interval can be set to 5, 10, 15, and 20 pixels. The corresponding stripe width and the computation time are shown in Figure 11. From Figure 11a, it can be seen that the ASW had no significant variations with the increase in interval space. The computation time, however, could be further reduced to smaller than 5 ms, as shown by Figure 11b. For the above experiments, it can be seen that the BPNN could get the width of the laser stripe accurately. More importantly, it can be extremely efficient, which makes the real time stripe width assessment possible. For the following experiments, the sampling interval was set as 10 pixels.

### 6.2. Adaptive Control for a Single Intersection Profile

The favorite cross section profiles of a stripe image should be the ones that have the maximum gray value and are not saturated as the profile that has the exposure time of t = 7 ms in Figure 3a. In this situation, the cross section profile can reach a favorite reliability [35]. The favorite width relies on the component properties of the LSLS like camera resolution, field of view, and width of the laser line. For the proposed sensor, the favorite width is computed from the profile and its width is about 10 pixels. Thus, the ideal width is $C_{\mathrm{avr}}^{\mathrm{set}}$ = 10 pixels, and the required range of stripe width can be set from $C_{\mathrm{avr}}^{\min}$ = 8 pixels to $C_{\mathrm{avr}}^{\max}$ = 12 pixels. The minimum gray threshold value was set as *θ*_g_ = 70.

**Figure 12:**
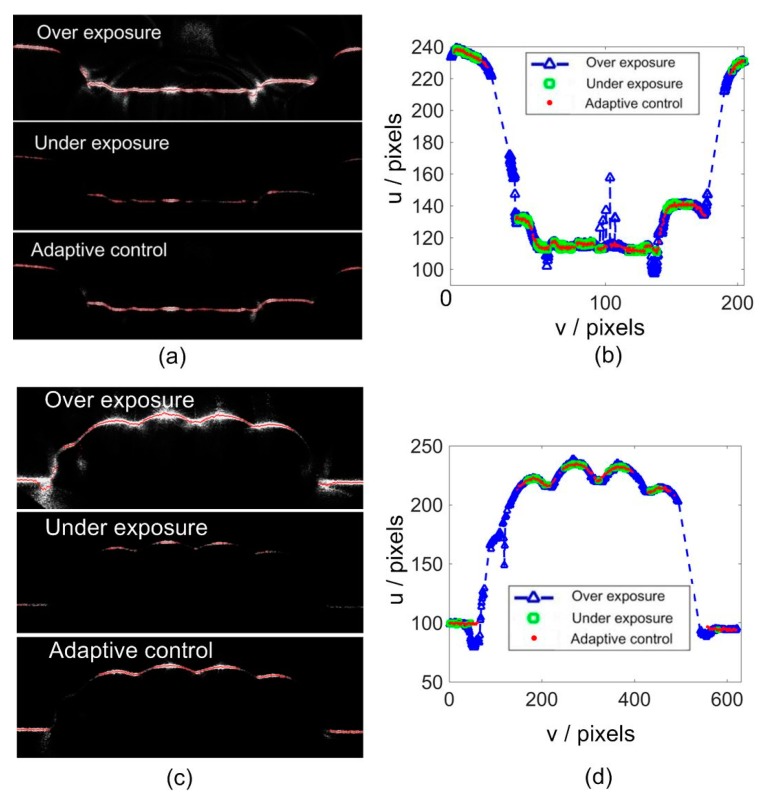
Comparison of center extraction results under different exposure conditions. (**a**,**c**) Stripe images; (**b**,**d**) center extraction results.

To analyze the effectiveness of the control method, two specific intersection profiles were examined. The first was the concave surface and the second was from a convex surface, as shown by Figure 12. For each one, three stripe images with different exposure times were captured and are illustrated by Figure 12a,c. In each figure, the stripe can be over exposed, under exposed, and adaptively controlled. The center line of each stripe is computed by the use of the gray gravity method [12]. For each cross section profile, 31 pixels were selected to compute the center point. The center extraction results are shown on the stripe images. For easy comparison, these lines are also plotted in Figure 12b,d, respectively. It can be seen that if the stripe image is over exposed, significant noises are introduced, especially at the corner regions. On the other hand, if the stripe is under exposed, many center points would be lost. When the adaptive control method is adopted, a more favorable stripe image and a center extraction result can be achieved. To remove the extremely over exposed cross section profiles, a maximum width threshold parameter was defined as *θ*_w_ = 25 pixels. If *C_v_* > *θ_w_*, the center point of this cross section will not be computed.

To further compare the center extraction results, each center line was fitted by use of the moving least squares method [12]. The fitting error can be used to evaluate the center extraction results. The absolute average error (AVR) and the root mean squared error (RMSE) for the three center lines are shown in Table 2. Thus, the smoothness of the stripe center lines can be effectively enhanced via the adaptive control.

### 6.3. Adaptive Control for Part Scanning

**Figure 13:**
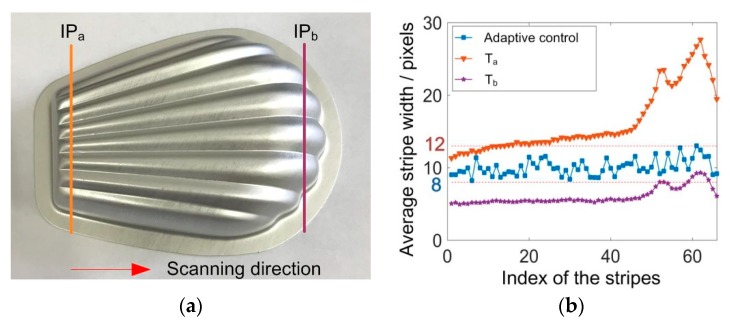
Measurement of a metal part. (**a**) The part and the specific intersection profiles; (**b**) the change of average stripe width during the scanning process.

To further demonstrate the advantages of our method, a stamped aluminum part was measured with the scanning direction shown in Figure 13a. When the laser plane intersected with the part at IP_a_, the adaptive control method was adopted and the ASW was modulated within the required range. The exposure time was recorded as T_a_. Similarly, when the laser plane intersected with the part at IP_b_, another optimal exposure time could be obtained as T_b_. For the traditional scanning process, the exposure time is unchanged. If T_a_ is selected as the constant exposure time, the ASW will change in accordance with the surface geometries, as shown by Figure 13b. At the right end of the part, the normal surface gets closer with the camera optical axis. The average stripe width increases significantly with some of the cross section profiles becoming over exposed. On the contrary, the exposure time of T_b_ would make most of the cross section profiles under exposed. Neither of them can guarantee a favorite ASW during the whole scanning process. When the proposed method is applied, the ASW can be adaptively controlled within the required range, which would benefit the stripe center extraction.

**Figure 14:**
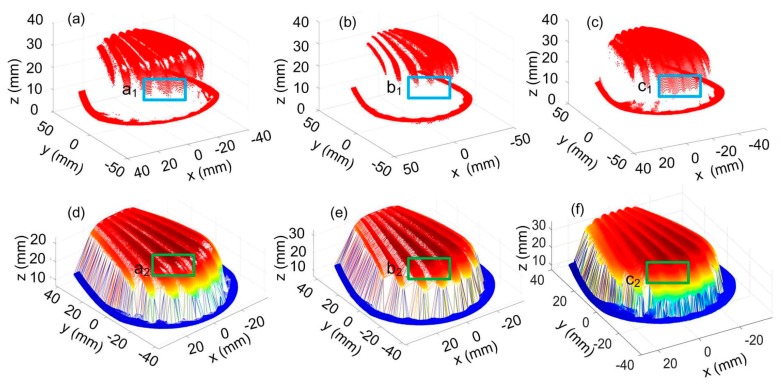
Point cloud data and the triangulated surface of a part with steep feature at different exposure conditions. (**a**,**d**) The exposure time is T_a_; (**b**,**e**) the exposure time is T_b_; (**c**,**f**) the exposure time is adaptively controlled.

Measurement results of the part are shown in Figure 14. Figure 14a–c are the point cloud data, and Figure 14d–f are the corresponding surfaces obtained via the Delaunay triangulation. Here, we mainly focused on the portions with a large slope denoted by a_1_, b_1_, c_1_ and the portions with large curvature denoted by a_2_, b_2_, c_2_, on the corresponding figures. It can be clearly seen that more points can be obtained with the adaptive control of the exposure time and the surface integrity can be enhanced.

**Figure 15:**
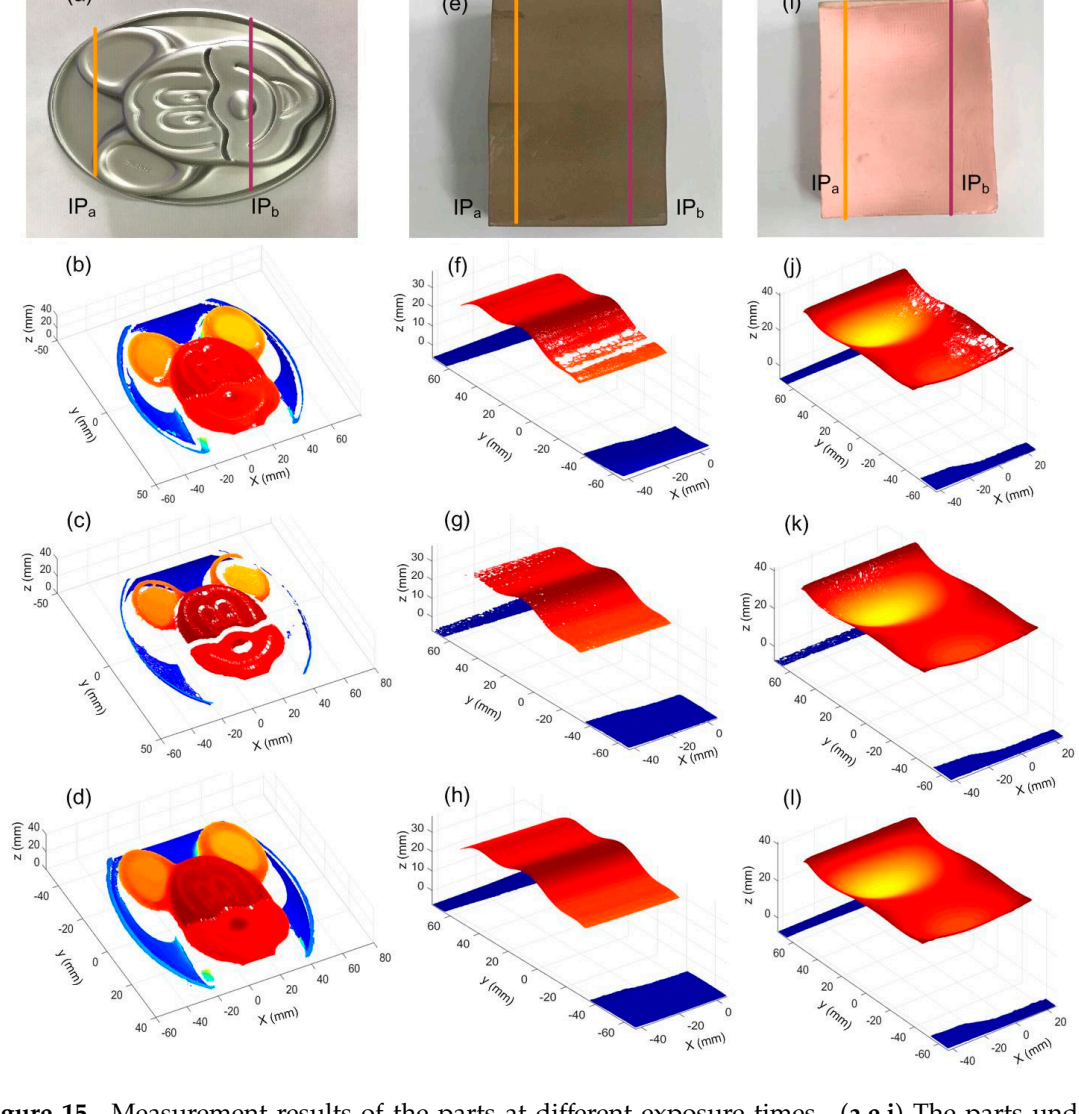
Measurement results of the parts at different exposure times. (**a**,**e**,**i**) The parts under inspection; (**b**,**f**,**j**) at a constant exposure time of T_a_; (**c**,**g**,**k**) at a constant exposure time of T_b_; (**d**,**h**,**l**) at adaptively controlled exposure time.

Other than the first part, three other parts with different surface properties were investigated. The first one was a complex ‘Mickey Mouse’ surface, the second was brown with a convex surface, and the last was pink with a concave surface, as shown in Figure 15. For comparative analysis, these parts were also scanned using different exposure times. The exposure time T_a_ and T_b_ can be obtained by using the same method as the part in Figure 13a. When constant exposure time is applied for the whole scanning process, the stripe can be over/under exposed due to the geometrical variation of the part. This would lead to missing data points and obvious holes on the reconstructed surfaces. The adaptive control of the exposure time, on the other hand, can effectively improve the measurement integrity.

### 6.4. Comparative Analysis of Effective Points

**Figure 16:**
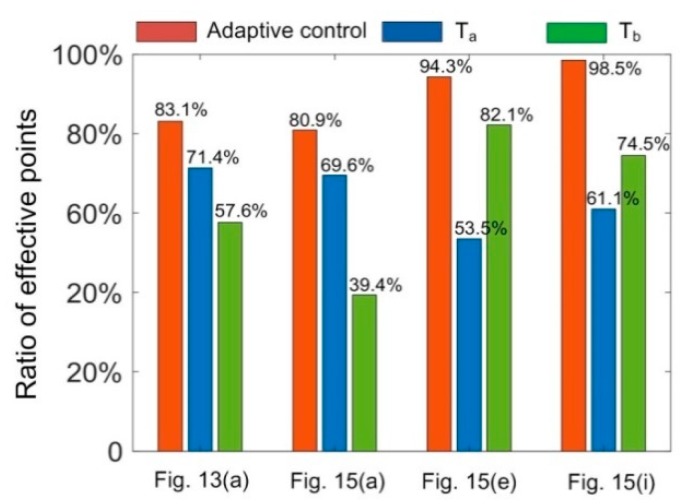
Ratio of effective points for the measurement of different parts at varied exposure conditions.

For each intersection profile, the number of ideal center points should be the same as the columns of the stripe image. One center point can be obtained for each column. However, the maximum gray value of a specific cross section profile can be smaller than the gray value threshold *θ*_g_ = 70 or the width of the cross section profile is larger than the presented value *θ*_w_ = 25 pixels. The center point of this cross section profile was not computed. The former case also included two situations: (1) The cross section profile was under exposed and (2) there was no stripe due to self-block of the part. Otherwise, one center point could be obtained for this column. Thus, the ratio of effective points can be defined as

(15) $$R_{\mathrm{eff}}=\frac{N_{\mathrm{eff}}}{V_{c}\cdot N_{\mathrm{val}}}\cdot 100\%$$

where *N*_eff_ is the number of effective points for the whole measuring process; *V*_c_ is the columns of each stripe image; and *N*_val_ is the number of effective stripe images. Ratio of effective points is shown in Figure 16. It can be seen that the *R*_eff_ value could be improved significantly for all cases when the exposure time was adaptively controlled during the measurement process.

### 6.5. Effective Analysis of Linear Iteration

**Figure 17:**
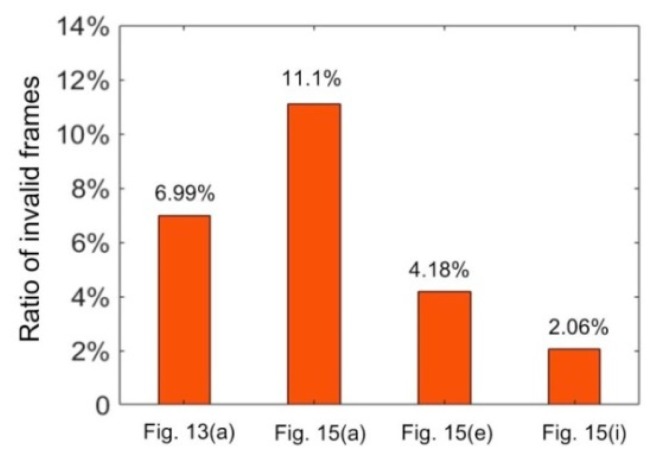
Ratio of invalid frames for the measurement of different parts.

In the measurement process, if the ASW locates within the required range of 8~12 pixels, this stripe image is used to compute the center points, which is called an effective image. Otherwise, the image is only used to estimate the exposure time of the next frame and is called an invalid stripe image. The invalid stripe images do not contribute to the 3D measured points. The ratio of ineffective image frames is defined by

(16) $$R_{\mathrm{in}}=\frac{N_{\mathrm{inval}}}{N_{\mathrm{total}}}\cdot 100\%$$

where *N*_inval_ is the number of invalid frames and *N*_total_ is the number of total frames in the measuring process. For the four parts above, the *R*_in_ values are shown in Figure 17. It can be seen that *R*_in_ relies on the part. For part Figure 13a, it had a large slope at the right end. For part Figure 15a, there was a steep feature at the center. These dramatic changes needed more iterative steps to achieve the required stripe width. Thus, both of them had relatively large *R*_in_ values. When the surfaces become smoother, the *R*_in_ value can be much smaller, like parts in Figure 15e,i. For the part in Figure 15i, the *R*_in_ value even reached 2.06% which means that the exposure time of the later image can be well estimated by the former one. This demonstrates that the proposed adaptive control method can improve the surface integrity without increasing the computation cost too much.

## 7. Conclusions

An adaptive control method was proposed to adjust the ASW via real-time tuning of the exposure time. To enhance the computing efficiency, the width of each stripe cross section profile was calculated by use of the BPNN. The reference width, which was used for the network training, was obtained by the GF method. The GF method is time consuming and not competent for real-time computation. The BPNN, on the other hand, can reduce the computation time to several milliseconds and makes real-time adaptive exposure control possible. To reveal the relationship between exposure time and ASW, several experiments were carried out. The results show that the exposure time vs. ASW curve approximately follows a linear manner. Therefore, a linear iteration method was brought out to adjust the camera exposure time. When the control method was applied, the ASW could be controlled within a required range and the quality of stripe image could be effectively improved. The measurement results of different parts showed that the control method could improve the surface integrity. The small proportion of ineffective images during the measurement process further validated the adaptive control method. It should also be noted that the dramatic changes of geometry would lead to varied exposure times. Thus, the sampling interval can be non-uniform. In further research, the scanning velocity may also be included in the adaptive control system to enhance the measurement quality.

## Figures and Tables

**Table 1:** Comparison of Gaussian fitting (GF) method with the back propagation neural network (BPNN) method.

		(a)	(b)	(c)	(d)	(e)	(f)
ASW (pixels)	GF	7.0721	8.0803	19.1964	6.2689	15.1558	4.6605
	BPNN	6.8747	7.9025	19.3939	6.0165	15.2291	4.4732
	Deviation	0.0279	0.0220	0.0103	0.0403	0.0048	0.0402
Time (s)	GF	1.2209	2.8378	1.7788	1.1309	2.8270	1.1546
	BPNN	0.0092	0.0136	0.0126	0.0099	0.0121	0.0110
	Relative percentage	0.75%	0.48%	0.71%	0.88%	0.43%	0.95%

**Table 2:** Center extraction errors of different stripe images (pixels).

	Error	Over Exposure	Under Exposure	Adaptive Control
Figure 12a	AVR	0.4857	0.4204	0.2771
	RMSE	0.7406	0.5584	0.3890
Figure 12c	AVR	0.4262	0.3693	0.2898
	RMSE	0.6664	0.5065	0.3932
